# The ironclad truth: how *in vivo* transcriptomics and *in vitro* mechanistic studies shape our understanding of *Neisseria gonorrhoeae* gene regulation during mucosal infection

**DOI:** 10.1093/femspd/ftx057

**Published:** 2017-05-17

**Authors:** Matthew R. Moreau, Paola Massari, Caroline A. Genco

**Affiliations:** Department of Integrative Physiology and Pathobiology, Tufts University School of Medicine, Boston, MA 02111, USA

**Keywords:** *Neisseria gonorrhoeae*, gene regulation, iron, transcriptome, mucosal infection

## Abstract

*Neisseria gonorrhoeae* is one of the most prevalent sexually transmitted infections worldwide. This obligate human pathogen has been extensively studied *in vitro*, where bacterial factors that are known to contribute to gonococcal disease and their regulation are relatively well defined. However, these *in vitro* experimental conditions only loosely replicate the host specific environment encountered by the bacteria *in vivo*. We recently reported on the complete gonococcal transcriptome expressed during natural human mucosal infection using RNA-seq analysis. Gene transcripts expressed *in vivo* (*in vivo* expressed factors) included genes encoding antibiotic resistance determinants, and a large number of hypothetical genes. A comparison of the gonococcal transcriptome expressed *in vivo* with the corresponding strain grown *in vitro* identified sets of genes regulated by infection, including those regulated by iron and the transcriptional regulatory protein Fur. We highlight here the role of Fur and gonococcal-specific regulatory processes important for infection and pathogenicity. We have determined that the genes controlled by Fur follow the same expression pattern *in vivo* as described previously *in vitro*, confirming Fur's regulatory role during infection. Collectively, these studies provide new insights into how bacterial fitness and pathogenicity are modulated during human mucosal infection.

## Teaser

This article reviews how combining transcriptomics on pathogens from naturally infected hosts and other mechanistic studies can improve our understanding the genes involved in bacterial pathogenesis and how they are regulated.

## INTRODUCTION

The Gram-negative obligate human pathogen *Neisseria gonorrhoeae* is the causative agent of the sexually transmitted infection gonorrhea, the second most common reportable disease in the USA. According to the Centers for Disease Control and Prevention (CDC) and the World Health Organization (WHO), there are approximately 800 000 new cases of gonorrhea in the USA and 106 million cases worldwide each year (World Health Organization 2012, 2016; Newman *et al.*^2015^; CDC 2016). *Neisseria gonorrhoeae* infects the genitourinary tract of men and women; however, the disease sequelae differ between genders (Edwards and Apicella 2004). In men, gonorrhea is defined by a marked infiltration of polymorphonuclear neutrophils (PMNs), resulting in symptomatic urethritis and epididymitis (World Health Organization 2012, 2016; Yu and Genco 2012a; Goire *et al.*^2014^). In women, the disease is more insidious, remaining asymptomatic and often resulting in spread of the infection to the ascending genitourinary tract (Walker and Sweet 2011; Islam *et al.*^2015^; Newman *et al.*^2015^). If left untreated in women, gonococcal infection can result in pelvic inflammatory disease, endometriosis, ectopic pregnancy and ultimately, infertility (Fichorova *et al.*^2001^; World Health Organization 2012, 2016; Islam *et al.*^2015^). Although the genitourinary tract is the primary site of infection in humans, *N. gonorrhoeae* can also infect extragenital sites such as the oropharynx and the rectum (Chan *et al.*^2016^).

A rising trend in incidence of *N. gonorrhoeae* has recently been reported, and this is expected to continue to rise, due in part to increased antibiotic resistance of the organism and the lack of a preventative vaccine (Unemo *et al.*^2012^; Unemo and Shafer 2014; Jerse, Bash and Russell 2015; Gottlieb *et al.*^2016^). A better understanding of *N. gonorrhoeae* pathogenic mechanisms during human mucosal disease would aide in development of novel therapeutics. There are a few *in vivo* models of gonococcal genital infection, but these present with several limitations. The human male urethral *N. gonorrhoeae* challenge model uses experimental infection of male volunteers as a model of urethritis (Cornelissen *et al.*^1998^; Biswas *et al.*^1999^). While studies in this model have expanded our understanding of acute infection, they do not address the consequences of long-term infection or infection specifically in women.

A mouse model of female gonococcal infection was developed in the late 1990s based on estradiol treatment of female mice, which allows for *N. gonorrhoeae* genitourinary tract infection in this otherwise resistant species (Jerse 1999; Jerse *et al.*^2011^). Although successful, this model represents a surrogate of female infection in humans in an artificial hormone condition. A more recent model based on generation of ‘humanized’ mice expressing the human carcinoembryonic antigen cell adhesion molecule (CEACAM) 1 and 5, required for *N. gonorrhoeae* opacity (Opa) proteins interactions with host cells overcomes some of these limitations (Schmitter *et al.*^2007^; Sadarangani, Pollard and Gray-Owen 2011). While this model has expanded the study of *N. gonorrhoeae* infection in both male and female mice without the use of estradiol (Gu *et al.*^2010^; Sintsova *et al.*^2015^), it does not mimic the unique environment of the human genital tract.

## GONOCOCCAL GENE EXPRESSION DURING INFECTION

**Figure 1.**
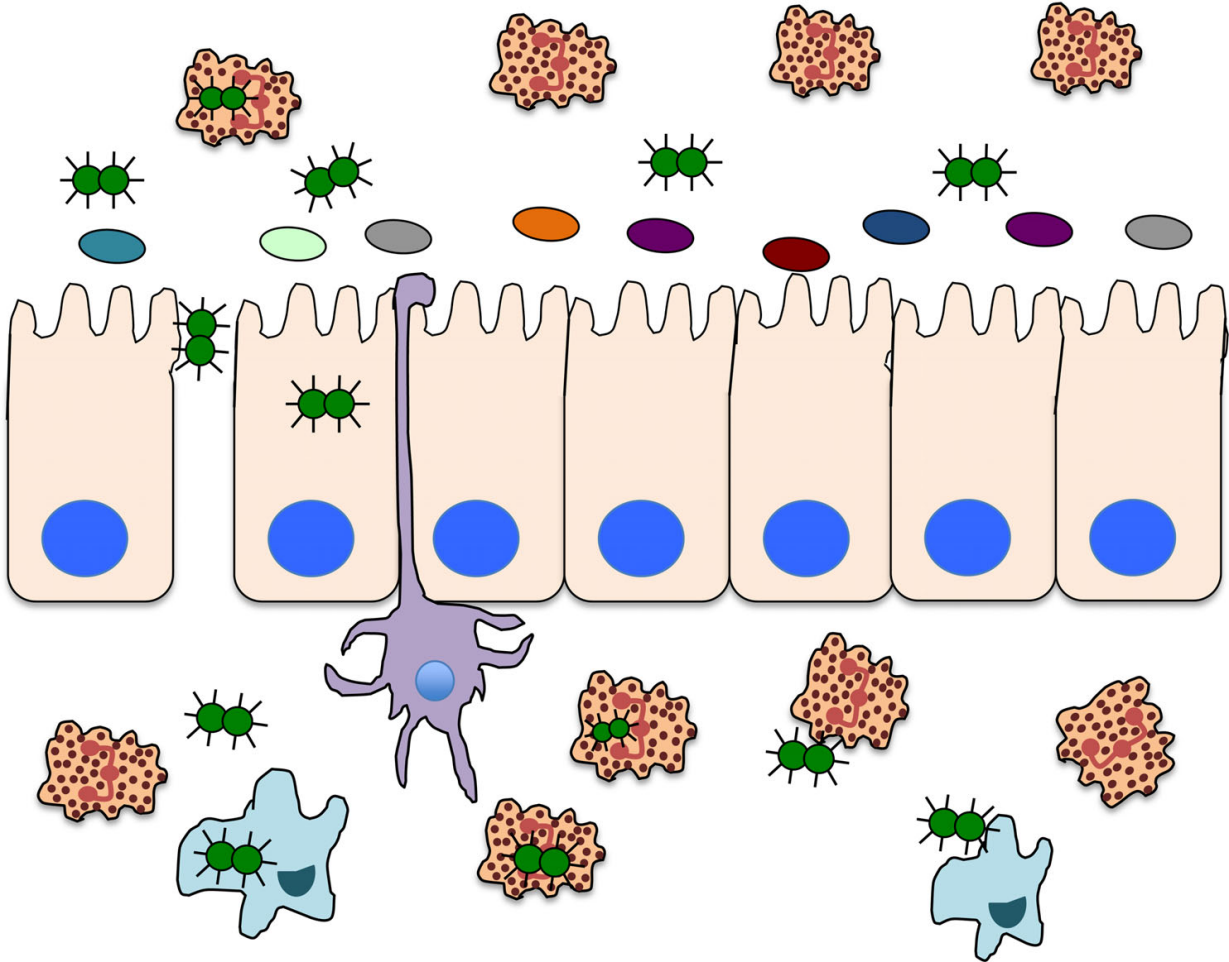
Mucosal environment during gonococcal infection. During infection, the gonococcus encounters several different host cells such as epithelial cells and PMNs, the latter of which function to engulf and degrade the organism. Gonococcal infection in men typically results in a robust immune infiltration by PMNs, whereas in women asymptomatic infection is common and *N. gonorrhoeae* exists as a biofilm. In both men and women, the genitourinary tract mucosa is an iron-deplete environment with most bioavailable iron being bound host proteins such as transferrin. The gonococcus also encounters other microbes during genital tract infection in men and women, although the microbiota in men is typically less robust and diverse than that of females. Gonococci have also been demonstrated to transverse the epithelial barrier.

Like other human pathogens, *Neisseria gonorrhoeae* has evolved mechanisms to adapt to the specific environments encountered during infection. However, the majority of studies aimed at characterizing this response of the gonococcus to different environmental stimuli have examined *N. gonorrhoeae* cultured in *in vitro* conditions that do not completely replicate the environment encountered in the human host (Biswas *et al.*^1999^; Grifantini *et al.*^2003^; Agarwal *et al.*^2008^). In the female genital tract, *N. gonorrhoeae* is exposed to an environment characterized by low pH, varying oxygen and iron levels, and the presence of additional microbes and host cells (Fig. 1) (O’Hanlon, Moench and Cone 2013; McClure *et al.*^2015^; Zozaya *et al.*^2016^). Free iron is scarce in the female host and is complexed to host iron-binding proteins including lactoferrin or transferrin, making the female genital tract iron deplete (Agarwal *et al.*^2008^). During menses, iron levels can rise and *N. gonorrhoeae* responds to these changing levels through regulation of gene expression (Anderson *et al.*^2001^; Jerse *et al.*^2002^; McClure *et al.*^2015^). During infection, the gonococcus encounters several different host cells such as epithelial cells and PMNs, the latter of which function to engulf and degrade the organism (Fig. 1). However, *N. gonorrhoeae* has been demonstrated to survive and replicate within epithelial cells and PMNs and to evade the antibacterial actions of PMNs (Criss and Seifert 2012). The gonococcus also encounters other microbes during genital tract infection in men and women (Weis and Nelson 2006; O’Hanlon, Moench and Cone 2013). Thus, the complexity of environmental signals during human mucosal infection is difficult to recapitulate by *in vitro* studies.

## GONOCOCCAL GENE EXPRESSION DURING NATURAL MUCOSAL INFECTION IN HUMANS

**Figure 2.**
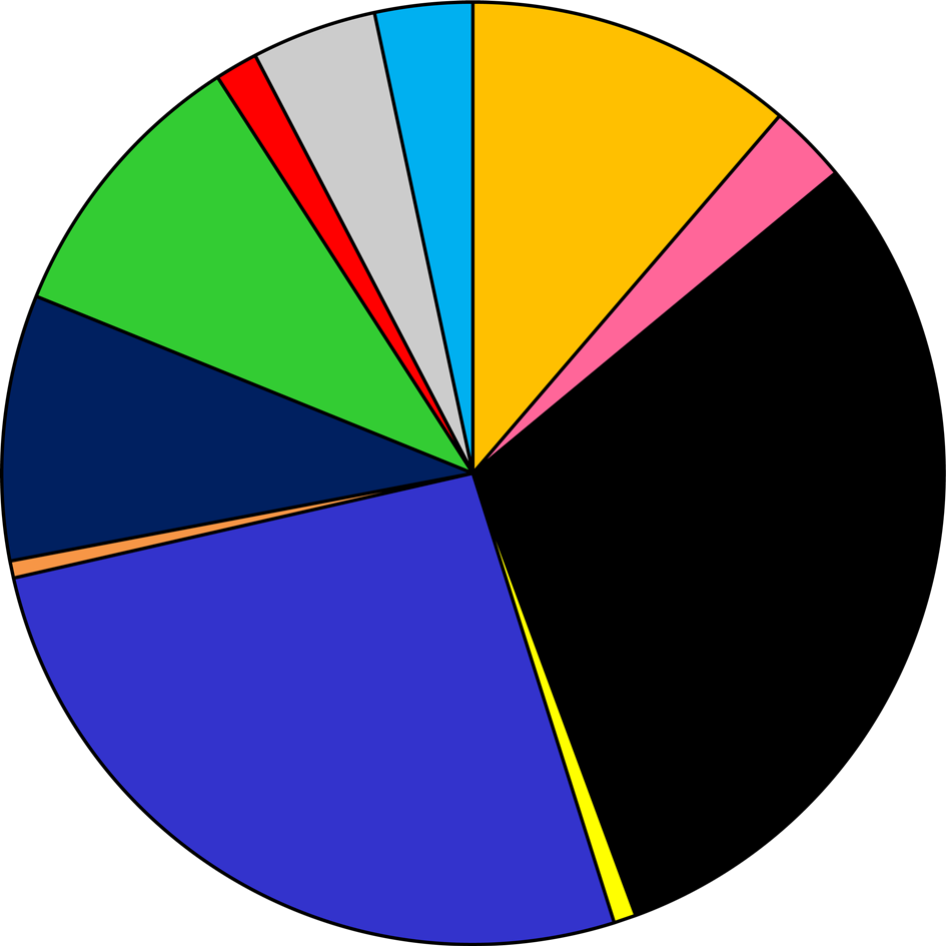
Gonococcal gene expression during mucosal infection in women. Approximately 1700 gonococcal genes are expressed during natural mucosal infection in women. Genes with RPKM values above 10 based on analysis with Rockhopper are grouped in 11 representative categories. The largest categories are hypothetical protein genes (517), indicated in black, and metabolism associated genes (447), which include general metabolism, energy, DNA and amino acid metabolism (blue). Expression-associated genes (192), including transcription, translation, rRNA synthesis and transcription factors, are indicated in goldenrod; sRNA genes (166) are in green; phage-associated genes (155), which include dsDNA, filamentous phages and transposase genes, are in dark blue; transport-associated genes (73) are in gray; tRNA genes (57) are in light blue. Host interaction-associated genes (46) include generally membrane-associated proteins such as adhesins, pilin biosynthesis and functions, lipoproteins, etc. and are indicated in pink; stress-associated genes (25) are in red; iron-associated genes (13) are in yellow and genes categorized as other (10) are in orange.

We previously reported on the expression of a subset of iron-regulated genes during human mucosal infection in men and women. These studies revealed that the gene encoding the transcriptional regulatory protein Fur (*fur)* and the Fur-regulated *tbpA/B* and *fbpA* genes were expressed in mucosal samples obtained from men and women with uncomplicated gonorrhea (Agarwal *et al.*^2005^). However, these studies were carried out using microarray and qRT-PCR analysis and were limited in their sensitivity and detection (Agarwal *et al.*^2005^, ^2008^). To define gonococcal global gene responses during human mucosal infection, we recently utilized RNA-seq analysis to define the complete gonococcal transcriptome in cervico-vaginal lavage samples from naturally infected female subjects. These studies revealed that 65% of the gonococcal genome was expressed during natural mucosal infection of the lower genitourinary tract in women. We detected expression of 1700 gonococcal genes which represented 22 functional categories and included large groups of hypotheticals, rRNA, sRNA, phage-associated and translation-related genes all (Fig. 2). Within these categories, we observed high expression of genes encoding antimicrobial efflux pumps, iron transport, phage, pilin, outer membrane and hypothetical proteins (McClure *et al.*^2015^).

The strains isolated from cervico-vaginal lavage specimens were also grown *in vitro* and resulting transcriptomes compared to those expressed during infection to define infection-specific expression profiles. This analysis established that a large portion of the gonococcal genome was regulated during mucosal infection relative to *in vitro* growth. Genes involved in DNA/RNA processing, genetic regulation, sugar uptake, amino acid processing and phage-associated proteins all displayed large differences in expression among these two datasets. Furthermore, expression levels of a group of gonococcal hypothetical genes were observed to vary between *in vivo* and *in vitro* conditions, leading to speculations that new metabolic or regulatory pathways may be discovered, as well as bacterial factors involved in virulence, evasion of host defense mechanisms (i.e. antibiotic resistance) and novel proteins that could represent new therapeutic targets (McClure *et al.*^2015^). Our analysis also revealed increased expression of Fur and iron-regulated genes during infection in women as compared to growth *in vitro*, suggesting that during infection of the genital tract in women, the gonococcus is exposed to an iron-deplete environment.

## TRANSCRIPTIONAL CONTROL BY FUR

**Figure 3.**
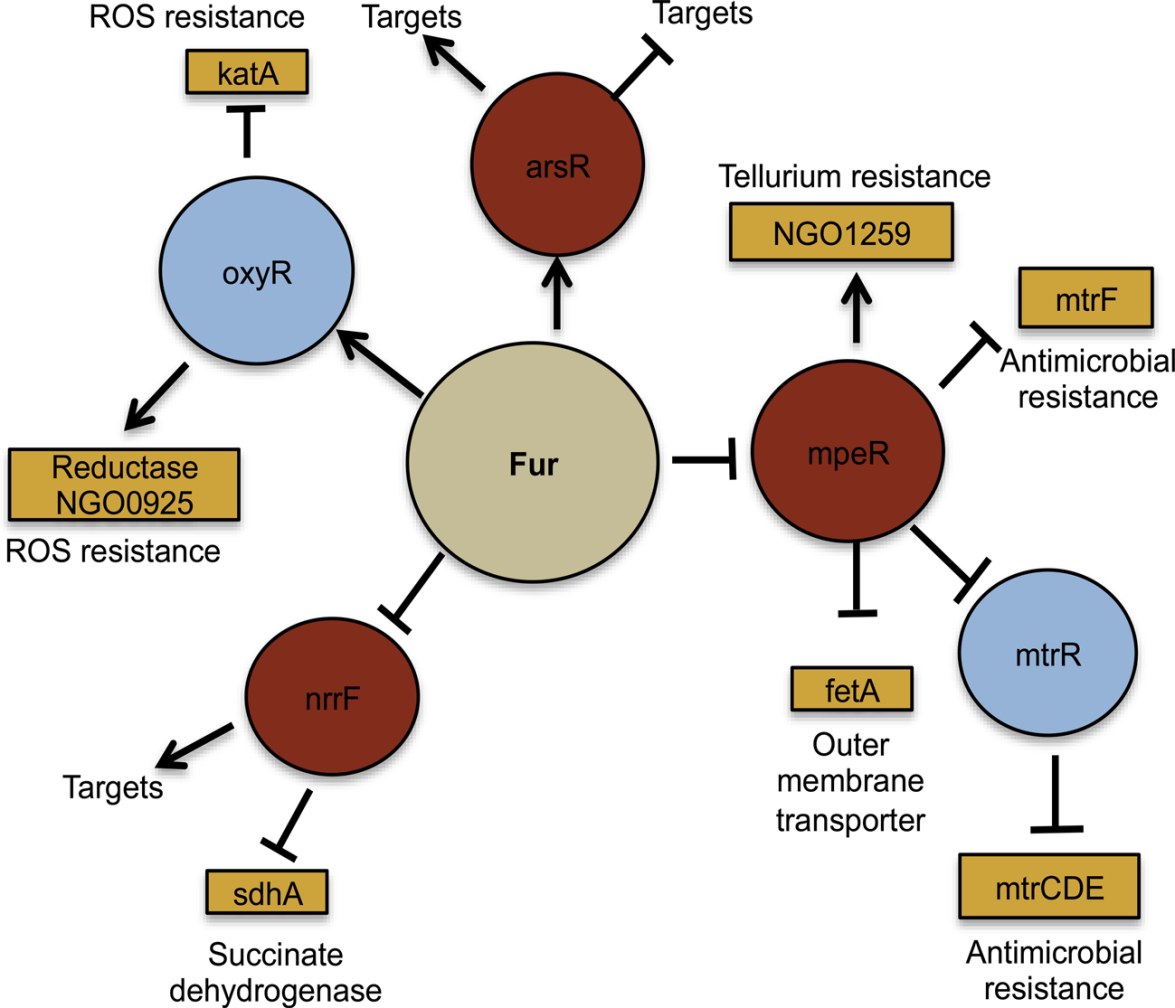
Fur interaction with regulatory proteins and sRNAs. Fur has been shown or hypothesized to interact with other regulatory proteins and sRNAs. Interactions between Fur (tan circle) and other regulators (light blue circles) are shown. Downstream targets of these regulators are shown and defined as indirect targets of Fur. Rust colored circles indicate regulators identified in our studies. Expression under iron-replete conditions is depicted.

The gonococcal Fur protein controls expression of iron homeostasis genes in response to intracellular iron levels (Fig. 3). This ensures a crucial balance between the requirement for iron, as an essential element for growth, and the avoidance of iron toxicity, which occurs via production of hydroxyl or peroxide radicals (Cornelissen *et al.*^1998^; Touati 2000; Seib *et al.*^2006^; Bartnikas 2012; Troxell and Hassan 2013). Classically, Fur binds directly to DNA sequences to inhibit transcription of downstream genes (Mellin *et al.*^2007^; Carpenter, Whitmire and Merrell 2009; Yu and Genco 2012a,b). One of the best-known examples involves transcriptional control of genes that scavenge iron from the host, including the transferrin binding proteins (*tbpAB*) and the ferric binding protein (*fbp*) (Gray-Owen and Schryvers 1996; Cornelissen *et al.*^1998^; Agarwal *et al.*^2005^; McClure *et al.*^2015^). In the absence of iron, Fur exists as an inactive monomer that becomes active when intracellular levels of iron are high allowing Fur to bind to promoter regions bearing the Fur box (Fig. 3) (Bagg and Neilands 1987; Troxell and Hassan 2013). While this interaction typically acts to block subsequent binding by RNA polymerase, Fur can also function as a transcriptional activator (Yu and Genco 2012a). Fur can also regulate genes indirectly by repressing a series of *trans* elements that regulate downstream targets. For example, sRNAs can act as repressors as is the case for the Fur repressed sRNA NrrF, which controls transcription of the *sdhC/A* genes (Fig. 3). Fur-mediated repression of NrrF results in increased translation of *sdhC/A* transcripts; thus, expression of functional SdhC/A proteins (succinate dehydrogenases involved in the TCA cycle) is indirectly activated by Fur (Ducey *et al.*^2005^; Agarwal *et al.*^2008^; Jackson *et al.*^2010^). Fur can also control additional regulatory proteins including ArsR, MpeR and OxyR (Fig. 3). OxyR is known to regulate resistance of the gonococcus to ROS (Seib *et al.*^2007^). MpeR targets genes involved in antimicrobial resistance including MtrR (Lee *et al.*^2003^; Warner *et al.*^2007^; Jackson *et al.*^2010^; Mercante *et al.*^2012^; Yu *et al.*^2016^). ArsR is a regulator of the *norB* gene involved in nitrous oxide reduction, as well as predicted to regulate NGO1411 and NGO1646 (encoding a hypothetical and phage-associated gene respectively) and has been shown to be important for intracellular survival in endocervical cells (Isabella *et al.*^2008^; Yu *et al.*^2016^).

### Fur regulation of regulatory and stress proteins in vitro

Superoxide and other oxide-containing effectors are stresses that *Neisseria gonorrhoeae* encounters within the human genital tract. In response to infection, epithelial cells and resident macrophages produce nitric oxide (NO) via AKT kinase activation and iNOS *in vitro* (Householder *et al.*^2000^; Seib *et al.*^2006^, ^2007^; Isabella *et al.*^2008^; Edwards 2010). To combat the effects of NO, *N. gonorrhoeae* expresses NorB, a NO reductase whose expression is repressed by the Fur-regulated ArsR regulatory protein (Isabella *et al.*^2008^). In iron-replete conditions *in vitro*, Fur functions as a transcriptional activator of *arsR*, a gene that is essential for survival within endocervical cells *in vitro* (Yu *et al.*^2016^).

Intrinsically, high levels of intracellular iron can react with the reduction and oxidation of NADH resulting in the formation of damaging reactive oxygen species (ROS) (Touati 2000; Seib *et al*., 2006, 2007). PMNs also express hydrogen peroxide (H_2_O_2_) and other oxidative species as antimicrobial compounds (Criss and Seifert 2012). The transcriptional regulator OxyR that is, in turn, regulated by active Fur regulates the ROS protection regulon; when Fur is active, it upregulates *oxyR* expression (Fig. 3). Thus, genes under the control of OxyR are also upregulated in iron-deplete environments (Seib *et al.*^2006^, ^2007^; Criss and Seifert 2012).

During growth under iron-deplete conditions, Fur is inactive and cannot repress the sRNA NrrF *in vitro*, which regulates target genes via post-transcriptional regulation (Mellin *et al.*^2007^; Jackson *et al.*^2013^). Thus, NrrF indirectly through Fur can regulate a series of genes, including the stress protein TdfF. TdfF is an essential gene for gonococcal intracellular survival *in vitro* in endocervical cells, and is downregulated in response to increased levels of NrrF (Jackson *et al.*^2013^). Fur-regulated TdfH is important for *N. gonorrhoeae* extracellular survival in the presence of PMNs. This is due to the zinc sequestration function exhibited by TdfH that can inactivate calprotectin in neutrophil extracellular traps (Jean *et al.*^2016^). In the female genitourinary tract mucosa, *N. gonorrhoeae* can form a biofilm, further advancing its transition toward anaerobic respiration (Steichen *et al.*^2008^, ^2011^; Phillips *et al.*^2012^). NrrF has also been shown to inactivate the *sdh* operon, which encodes an iron-containing enzyme involved in the TCA cycle aspect of aerobic respiration. In the anaerobic and iron-limiting conditions observed *in vivo*, downregulation of this operon is predicted to enhance *N. gonorrhoeae* survival (Jackson *et al.*^2013^; Jean *et al.*^2016^).

Epithelial cells and PMNs expose *N. gonorrhoeae* to host-derived antimicrobials that serve as primary immune defenses such as fatty acids, defensins and hydrogen peroxide (Quayle 2002; Lee *et al.*^2003^; Johnson *et al.*^2015^). Most charged and non-charged antimicrobial compounds are exported from gonococci via efflux pumps such as FarAB and MtrCDE (Rouquette, Harmon and Shafer 1999; Lee *et al.*^2003^). Expression of the genes encoding these efflux pumps is tightly controlled due to their important role in the development of resistance to antimicrobial and antibiotic stress. Fur plays an integral role in regulation of the antimicrobial resistance efflux pump system encoded by the *mtr* locus via upstream regulation of the MtrR transcriptional regulatory protein (Lee *et al.*^2003^; Yu *et al.*^2016^). During growth under iron-deplete conditions, such as those observed during human mucosal infection, Fur is inactive and as such expression of MpeR, the transcriptional regulator of the *mtrR*, is increased resulting in depression of *mtrR* expression (Fig. 3). The inhibition of *mtrR* expression results in expression of the *mtrCDE* operon that encodes the multidrug efflux pump proteins (Mercante *et al.*^2012^). Interestingly, our analysis of gonococcal gene expression profiles in cervico-vaginal lavage specimens revealed that both *mtrR* and *mtrCDE* expression levels were similar to the levels expressed during *in vitro* culture in the presence of iron (McClure *et al.*^2015^). These observations lead us to speculate that loss in regulation of these genes during growth *in vivo* could results from promoter allele changes altering the interaction between the repressor, MtrR, and the *cis* elements in the promoter. Indeed, allele changes in these operons have been reported to be involved in increases in antimicrobial resistance as well as intracellular survival in the gonococcus (Warner *et al.*^2007^; Kirkcaldy, Kidd and Weinstock 2013; Unemo and Shafer 2014; Grad *et al.*^2016^).

### Fur control of gonococcal factors involved in host–pathogen interactions

During initial mucosal infection, the gonococcus interacts with epithelial cells via the Opa proteins and their cognate receptors, CEACAM 1, 3, 5 and 6 on the epithelial cell surface. This results in downstream signaling that favors subsequent gonococcal invasion of epithelial cells (Schmitter *et al.*^2007^; Sadarangani, Pollard and Gray-Owen 2011; Tchoupa, Schuhmacher and Hauck 2014; Sintsova *et al.*^2015^). Fur has been demonstrated to bind to and repress the gonococcal *opa* promoter during growth of the gonococcus under iron-replete conditions *in vitro* (Sebastian *et al.*^2002^). In concert with these observations, Opa-negative strains are predominantly recovered from infected female subjects during menses, when iron is abundant in the lower genitourinary tract (Sebastian *et al.*^2002^; Folster *et al.*^2009^; Jackson *et al.*^2010^; Sadarangani, Pollard and Gray-Owen 2011; Yu and Genco 2012b).

Intracellular survival of *N. gonorrhoeae* within endocervical cells *in vitro* as well as colonization within the female mouse model is partially mediated by the expression of a Fur-controlled phage repressor *npr* (*Neisseria* phage repressor) (Daou *et al.*^2013^). Despite the ability of Fur to bind the common promoter between *npr* and the four genes immediately downstream, we demonstrated that expression of *npr* is not regulated by iron or Fur in contradiction to previous studies (Ducey *et al.*^2005^; Jackson *et al.*^2010^; Daou *et al.*^2013^).

Opa proteins also mediate interaction of the gonococcus with PMNs (Johnson *et al.*^2015^). PMNs are the primary immune cells observed during *N. gonorrhoeae* infection in symptomatic males and females (albeit to a lesser extent in females compared to males) and the gonococcus has adapted mechanisms that enable the organism to survive within PMNs (Edwards and Apicella 2004). In women, *N. gonorrhoeae* infection is also associated with biofilm formation, protecting the organisms from immune cells such PMNs (Fig. 1). Evidence suggests that iron is more replete within a biofilm based on expression of iron-responsive gene products that are upregulated during growth in iron-deplete conditions, such as FetA and TbpA/B (Phillips *et al.*^2012^). Eventually, however, some bacteria escape the biofilm and enter into the iron-deplete luminal space where they make contact with PMNs (Steichen *et al.*^2011^; Phillips *et al.*^2012^). Survival after internalization of *N. gonorrhoeae* into PMNs *in vitro* depends on Opa expression. Expression of Opa proteins results in efficient clearance mediated by serine protease activity (Ball and Criss 2013; Johnson and Criss 2013; Johnson *et al.*^2015^).

## OUTLOOK/CONCLUSIONS

*Neisseria gonorrhoeae* is one of the most common bacterial sexually transmitted infections worldwide (World Health Organization 2016). The incidence of infections has increased due in part to the continuing evolution of bacterial mechanisms of antimicrobial resistance and the asymptomatic nature of the disease which results in increased transmission. The lack of a good experimental model that can replicate the various microenvironmental conditions *N. gonorrhoeae* is exposed to within the human host has led to a significant gap in our understanding of its pathogenic strategies during natural mucosal infection. Transcriptome studies of gonococci during natural human mucosal infection reported by our group in combination with *in vitro*-based regulatory studies have shown that several regulatory circuits control *N. gonorrhoeae* genes important for colonization and survival. We demonstrated that the Fur transcriptional regulatory protein extends not only to iron-regulated genes, but also to genes involved in a number of other regulatory networks. All of these studies show that the general trend of activation or repression of genes by Fur observed *in vitro* is mimicked *in vivo*, further showing the importance of Fur as a central regulator during infection. A comparison of the gonococcal transcriptome expressed *in vivo* to the corresponding strain grown *in vitro* revealed increased expression of genes involved in iron transport including *tbpAB* and *fbpABC in vivo* (Agarwal *et al.*^2005^, ^2008^; McClure *et al.*^2015^). As TbpA and TbpB have been considered potential gonococcal vaccine candidates, defining the details of their regulation *in vivo* compared to *in vitro*, may be beneficial for future characterization of the therapeutic potential of these proteins. In addition, it is possible that other regulated factors, among both the known and hypothetical gene products revealed by our transcriptome studies, may represent novel targets for therapeutic and preventive strategies, i.e. new antimicrobial factors or vaccine candidates.

Current studies are focused on gonococcal transcriptional profiles expressed during infection in men. These studies have revealed a divergence in both the disease presentation in men and women, and gonococcal transcriptional programming*.* Initial results suggest that there is significant overlap in genes expressed by the gonococcus during mucosal infection in both men and women, including genes related to general metabolism and transport. Distinct gene sets expressed during infection in men were enriched for genes encoding host interaction and membrane-associated proteins. In contrast, distinct gene sets expressed during infection in women were enriched for iron and DNA metabolism genes (Nudel *et al.*, unpublished). Further analysis of these unique data sets obtained from infected male and female subjects will define gonococcal adaptions during natural mucosal infection, a long-term goal of our studies. Taken together, these studies offer powerful new insights into the pathobiology of *N. gonorrhoeae* and will not only lead to a better understanding of the mechanisms of gene regulation employed by *N. gonorrhoeae* during infection, but ultimately will allow for the identification of novel virulence factors and consequently expand the potential for preventive and therapeutic strategies against *N. gonorrhoeae* infection.

***Conflict of interest.*** None declared.
